# Impact of CYP2C19 Genotype on Efficacy and Safety of Clopidogrel-based Antiplatelet Therapy in Stroke or Transient Ischemic Attack Patients: An Updated Systematic Review and Meta-analysis of Non-East Asian Studies

**DOI:** 10.1007/s10557-023-07534-0

**Published:** 2023-12-01

**Authors:** Sarah Cargnin, Federica Ferrari, Salvatore Terrazzino

**Affiliations:** 1 Department of Health Sciences, Università del Piemonte Orientale (UPO), Novara, Italy; 2 Dept of Brain and Behavioral Sciences, University of Pavia, Pavia, Italy; 3 Department of Emergency Neurology and Stroke Unit, IRCCS Mondino Foundation, Pavia, 27100 Italy; 4 Department of Pharmaceutical Sciences, University of Piemonte Orientale A. Avogadro. Largo, Donegani 2, Novara, 28100 Italy

**Keywords:** Ischemic Stroke, Transient Ischemic Attack, Clopidogrel, CYP2C19, meta-analysis

## Abstract

**Purpose:**

Inconclusive and limited results have been reported on the clinical utility of CYP2C19 genotyping in stroke/TIA patients of non-East Asian ancestries. We herein performed an updated systematic review and meta-analysis to quantitatively estimate the association of CYP2C19 loss-of function (LOF) status with efficacy and safety of clopidogrel-based antiplatelet therapy in non-East Asian patients affected by stroke or TIA.

**Methods:**

A comprehensive search was performed up to July 2023 using PubMed, Web of Knowledge, and Cochrane Library databases. The clinical outcomes investigated were stroke, composite vascular events and bleeding. Pooled estimates were calculated as risk ratios (RR) with 95% CI using the Mantel– Haenszel random-effects model. The quality of evidence was assessed using the GRADEpro tool.

**Results:**

A total number of 1673 stroke/TIA patients from 8 non-East Asian studies, published between 2014 and 2022, were included in the systematic review. Clopidogrel-treated carriers of CYP2C19 LOF alleles were found at increased risk of stroke compared to non-carriers (RR: 1.68, 95%CI: 1.04–2.71, P = 0.03). However, no significant association was observed with the risk of composite vascular events (RR: 1.15, 95%CI: 0.58–2.28, P = 0.69) or bleeding (RR: 0.84, 95%CI: 0.38–1.86, P = 0.67). Similarly, European ancestry patients carrying CYP2C19 LOF alleles displayed a higher risk of stroke (RR: 2.69 (1.11–6.51, P = 0.03), but not of composite vascular events or bleeding.

**Conclusion:**

The present updated meta-analysis provides moderate quality evidence of association between CYP2C19 LOF alleles and an increased risk of stroke in non-East Asian patients with stroke/TIA after receiving clopidogrel therapy. Further large pharmacogenetic studies are still warranted to corroborate these findings.

**Supplementary Information:**

The online version contains supplementary material available at 10.1007/s10557-023-07534-0.

## Introduction

Stroke affects more than 7 million of people per year worldwide and currently represents the second-leading cause of death globally [1, 2]. Approximately 80% of all strokes are ischemic and in 15–30% of cases they are preceded by a transient ischemic attack (TIA) [3–5]. Patients surviving a stroke are at an increased risk for subsequent serious vascular events and should receive secondary prevention measures, including antithrombotic therapy, improvements in lifestyle and management of vascular risk factors, primarily hypertension, dyslipidemias, and diabetes [6–8].

Clopidogrel-based antiplatelet therapy represents the mainstay treatment for secondary stroke prevention alongside aspirin. However, one-third to one-half of patients develop recurrent stroke or other clinical ischemic events while on antiplatelet therapy, with the highest recurrence risk observed within the first year of treatment [9, 10]. Clopidogrel resistance is thought to contribute to stroke recurrence risk, and factors including non-compliance, inadequate dosing, as well as interactions with other drugs, are common causes of poor antiplatelet therapy efficacy [11]. Clopidogrel resistance has been also ascribed to polymorphic variants of CYP2C19, which is recognised as the most important pharmacogene for clopidogrel [12]. The CYP2C19 gene is a member of the cytochrome P450 gene family involved in the bioactivation of clopidogrel that, once orally administered, requires a two-step biotransformation to generate an active thiol metabolite that prevents platelet aggregation by the irreversibly binding to the adenosine diphosphate (ADP) P2Y12 [13, 14]. It is well known that carriers of CYP2C19 loss-of-function alleles (mainly *2, *3, or *8) have an impaired pharmacodynamic response to clopidogrel compared to normal or rapid CYP2C19 metabolizers [15].

There is convincing evidence of an association between CYP2C19 genotype and clinical outcomes in stroke or TIA patients treated with clopidogrel. Indeed, results of a large meta-analysis of 15 studies [16], which has been published in 2017, highlighted that reduced-function CYP2C19 allele carriers have an increased risk of stroke following TIA or ischemic stroke when treated with clopidogrel compared to non-carriers (RR: 1.92, 95%CI: 1.57–2.35, P < 0.00001). Yet, of the 15 studies identified, 11 were from China, 1 was from South Korea and only 3 studies included patients with European ancestry [17–19]. The subsequent subgroup analysis based on patient’s ethnic origin revealed a firm association of CYP2C19 genotype with risk of stroke in patients of East-Asian ancestry (RR: 1.93, 95%CI: 1.55–2.39, P < 0.00001), while a large uncertainty of the pooled estimate was found in patients with European ancestry (RR: 2.46, 95%CI: 1.06–5.72, P < 0.04). These and other more recent data [20, 21] have provided consistent results within the East-Asian population, while the value of CYP2C19 genotype as pharmacogenetic determinant in other populations of patients with stroke or TIA is still unclear. Therefore, in the present study, we conducted an updated systematic review and meta-analysis to clarify the association of CYP2C19 genotype with efficacy and safety of clopidogrel-based antiplatelet therapy in non-East Asian patients with stroke or TIA.

## Methods

### Search and Inclusion/Exclusion Criteria

This systematic review was conducted according to the PRISMA guidelines [22] and adheres to the PRISMA checklist, which is shown in Supplementary Table 1. The protocol was prospectively registered on the International Platform of Registered Systematic Review and Meta-analysis Protocols (INPLASY) (Registration No. 202,370,067, https://inplasy.com/inplasy-2023-7-0067). PubMed, Web of Knowledge and Cochrane Library databases were systematically searched from inception up to 1st July 2023 for identification of potential eligible studies using the Boolean combination of the following key terms: (clopidogrel OR Plavix) AND (stroke OR “cerebrovascular accident” OR “cerebrovascular disorder” OR “cerebral infarction” OR “transient ischemic attack” OR “TIA” OR “transient cerebral ischemia”) AND CYP2C19.

Studies were included if they met the following eligibility criteria: (1) non-East Asian studies including patients with stroke or TIA treated with clopidogrel monotherapy or clopidogrel plus aspirin; (2) studies reporting comparison of clinical outcomes between carriers and non-carriers of CYP2C19 LOF alleles; (3) sufficient data to estimate the rate of at least one of the following outcomes: (a) stroke, (b) composite vascular events of stroke, myocardial infarction, and vascular death, (c) any bleeding. Studies were excluded if they meet one or more of the following criteria: (1) studies conducted exclusively in Eastern Asian populations; (2) studies including patients treated with clopidogrel for conditions other than stroke or TIA (e.g. patients treated with clopidogrel due to coronary heart disease); (3) studies with less than 10 eligible cases; (4) article written in a language other than English. When relevant data could not be extracted from a potential eligible study, the corresponding author was contacted via email. The study was excluded if the corresponding author did not respond to the email or did not provide the data requested for calculation of risk estimates. Reference lists of eligible studies were also reviewed to identify additional relevant reports. All studies were independently analysed by two reviewers (S.C and S.T) and discrepancies were resolved by discussion and consensus with a third reviewer (F.F).

### Data Extraction

From each study included in the systematic review, the following data were extracted: the first author’s last name, year of publication, study location and design, diagnosis of participants (stroke and/or TIA), patients’ ischemic stroke subtype according to the TOAST criteria [23], total number of patients involved, mean age of patients, percentage of women, the outcomes investigated, duration of patients’ follow-up, clopidogrel dose and whether administered alone or in combination with aspirin, CYP2C19 variant allele analysed, number of events in both carriers and noncarriers of CYP2C19 LOF alleles for each of the investigated outcome. Other data extracted from each study included number of patients of East Asian ancestry, number of patients with atrial fibrillation, number of patients with other concomitant antithrombotics or other concomitant drugs including PPIs and antidepressants, and number of smokers. In each study, clopidogrel-treated patients were classified as carriers of reduced-function CYP2C19 alleles (heterozygotes, homozygotes or compound heterozygotes for CYP2C19*2, CYP2C19*3 or CYP2C19*8) or non-carriers (wild-type or CYP2C19*17 carriers). Whenever available, genotype data were extracted also for patients of European ancestry from studies including patients with different ethnic origin. All studies have been independently analyzed by two reviewers (S.C. and S.T.) and any discrepancies were reconciled via consensus or arbitration by a third reviewer (F.F.).

### Assessment of Study Quality and Quality of Evidence

Methodological quality of studies included in the systematic review was independently assessed by two authors (S.C. and S.T.) using the Newcastle–Ottawa scale (NOS) (available at: https://www.ohri.ca//programs/clinical_epidemiology/oxford.Asp) and disagreements were resolved through discussion and consensus. The NOS contains eight items categorized into three dimensions including selection criteria of participants, comparability, and exposure. The NOS ranges between zero up to nine stars, with a higher number of stars indicating a higher methodological study quality. The overall methodological quality of each study was determined according to the total NOS score achieved: low quality (NOS score, 0–3), medium quality (NOS score, 4–6) and high quality (NOS score, 7–9). Rating of the overall quality of evidence for each outcome investigated was assessed by two authors (S.C. and S.T.) using the GRADEpro Tool (freely available at https://www.gradepro.org). Any discrepancies between authors were resolved through discussion.

### Statistical Analysis

The effect measure of interest was the risk ratio (RR), which was expressed as point estimate with 95% confidence interval (CI) for the comparison of reduced-function alleles carriers of CYP2C19 (intermediate metabolizers or poor metabolizers) vs. non-carriers (wild-type, rapid or ultrarapid metabolizers). Whenever data available, RR (95% CI) was also calculated for carriers of 1 CYP2C19 LOF allele in comparison with non-carriers and for carriers of 2 CYP2C19 LOF alleles vs. non-carriers. Pooled RRs and 95% CIs were calculated by RevMan 5.3 software using the Mantel–Haenszel random-effects model, as this method provides a more conservative estimate in case of potential heterogeneity across the studies [24]. Between-study heterogeneity was assessed by the chi-square–based Cochran’s *Q* statistic (significant for P < 0.10) and the I^2^ index (range, 0-100%), which quantifies heterogeneity independently of the number of studies. Leave-one-out sensitivity meta-analyses were performed to assess robustness of the estimated effect sizes by excluding individual results one at a time and recalculating the pooled RR estimates for the remaining studies. In addition, subgroup analyses in the European subgroup were conducted when data were available in at least two independent studies, as well as sensitivity analyses based on studies with at least 1 year of patients’ follow-up. The ProMeta version 2 software (INTERNOVI di Scarpellini Daniele s.a.s., Cesena FO, Italy) was used for performing leave-one-out sensitivity meta-analyses and for assessment of publication bias, which was statistically evaluated by the Egger’s test (significant for P < 0.10). The statistically significant threshold for all pooled analyses was set at *P* < 0.05.

## Results

### General Characteristics and Quality of Included Studies

**Figure 1:**
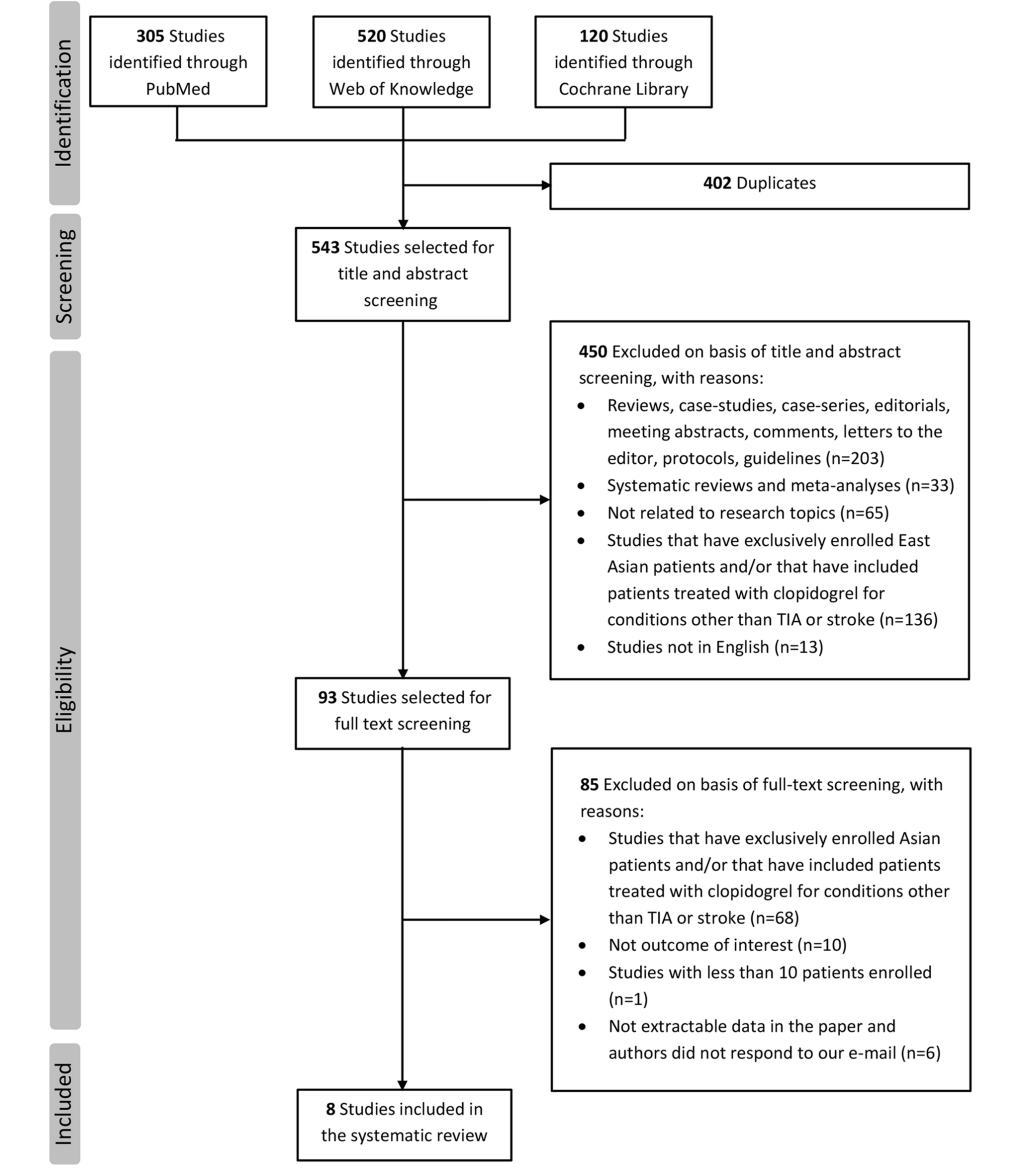
Flowchart of literature search and selection process of eligible studies

The flowchart of literature review and the screening process with reasons for study exclusion is shown in Fig. 1. Initially, a total of 945 studies were identified by searching Web of Knowledge, PubMed and Cochrane library databases. Of these, 402 hits were excluded for duplication and 535 additional reports were excluded for not meeting the inclusion criteria. Finally, 8 articles published between 2014 and 2022 were included in the systematic review of the effect of CYP2C19 genotype on clinical outcomes of non-East Asian patients with stroke or TIA receiving clopidogrel therapy [17–19, 25–29]. Main characteristics of the eight identified studies are shown in Table 1, and additional study characteristics are shown in Supplementary Table 2. Among these, 3 studies included patients from European countries [26, 27, 29], 2 from USA [17, 19], one from Turkey [25] and 2 were multiregional studies including patients from different non-Asian countries [18, 28]. One study was a post hoc analysis of RCT [18], while the remaining 7 were cohort studies. Six studies [17–19, 25, 28, 29] enrolled both stroke and TIA patients and 2 included patients with ischemic stroke only.^26,27^ Overall, 1673 cases were included in the present systematic review, ranging from a minimum of 43 in the study of Spokoyny et al. [17] to a maximum of 522 in the report of McDonough et al. [18], with a percentage of women varying between 36.7% [19] and 58.8% [25]. In three studies [25, 27, 29], clopidogrel was administered as monotherapy, in other three studies it was administered in combination with aspirin [18, 19, 28], while for two studies [17, 26] the clopidogrel-based regimen was not specified. The CYP2C19 LOF alleles analysed were *2 and *3 in 4 studies [19, 25, 28, 29], only *2 in 3 studies [18, 26, 27], while in one study [17] the CYP2C19 variant alleles analysed were not specified. As regard to study’s methodological quality (Table 1), the NOS scores ranged from 4 to 9, with an average score of 7.5, indicating an overall high methodological quality of studies included in the systematic review. Individual item scores achieved by each study are shown in Supplementary Table 3.

**Table 1:** Main characteristics of studies included in the systematic review

First author [Ref] (year)	Country	Participant	Ischemic stroke subtype according to the TOAST criteria, N (%)	Patients enrolled in the PGS, N (% of women)	Age, mean ± SD or median (IQR)	Outcome ^§^	Follow-up	Clopidogrel- based regimen (dose)	CYP2C19 variant alleles	NOS score
Spokoyny I et al. [17] (2014)	USA	Stroke or TIA	NR	43 (46.6)	69.6 ± NR	A	NR	NS	NR	4
Sen HM et al. [25] (2015)	Turkey	Stroke or TIA	NR	51 (58.8)	66.4 ± 9.6	A	At least 1 year	clopidogrel monotherapy (75 mg/die)	*2, *3	6
McDonough CW et al. [18] (2015)	USA, Canada, Chile, Ecuador, Mexico, Peru, Spain	Stroke or TIA	NR	522 (38.1)	62.5 ± 10.5	A, B	3.4 (0–8.2) years	clopidogrel (75 mg/die) + aspirin (325 mg/die)	*2, *17	9
Hoh BL et al. [19] (2016)	USA	Stroke or TIA	NR	188 (36.7)	67.0 ± 12.2	A, C	1 year	clopidogrel (NR) + aspirin (NR)	*2, *3, *8, *17	8
Tornio A et al. [26] (2018)	UK	Stroke	NR	94 (38.3)	74 ± NR	C	2 years	NS	*2	9
Tomek A et al. [27] (2018)	Czech Republic	Stroke	1: 61 (46.9) 2: 7 (5.4) 3: 10 (7.7) 4: 0 (0.0) 5: 52 (40.0)	130 (40.0)	64.5 ± 13.9	B, C	14.9 ± 14.0 months	clopidogrel monotherapy (NR)	*2, *17	8
Meschia JF et al. [28] (2020)	USA, Australia, Canada, Finland, France, Mexico, Germany, New Zealand, Spain, UK	Stroke or TIA	NR	457 (43.3)	63 (53–72)	A, B, C	3 months	clopidogrel (loading dose: 600 mg at day 1 followed by 75 mg/die) + aspirin (50–325 mg/die)	*2, *3, *17	9
Minderhoud C et al. [29] (2022)	The Netherlands	Stroke or TIA	1: 36 (19.1) 2: 10 (5.3) 3: 33 (17.6) 4: 8 (4.3) 5: 101 (53.7)	188 (42.0)	72.4 ± 8.8	A	3 months	clopidogrel monotherapy (75 mg/die)	*2, *3	7

* Subtype 1, large-artery atherosclerosis; subtype 2, cardioembolic; subtype 3, small vessel occlusion; subtype 4, other determined aetiology; subtype 5, undetermined cause

^§^ Outcome A, stroke; outcome B, any bleeding; outcome C, composite vascular events of stroke, myocardial infarction, and vascular death

Abbreviations: IQR, interquartile range; LOF, loss-of-function; NOS, Newcastle-Ottawa Scale; NR, not reported; NS, not specified if clopidogrel was administered alone or in combination with aspirin; PGS, pharmacogenetic study; SD, standard deviation; TIA, transient ischemic attack; TOAST, Trial of Org 10172 in Acute Stroke Treatment; USA, United States of America; UK, United Kingdom.

### Quantitative Data Synthesis

**Figure 2:**
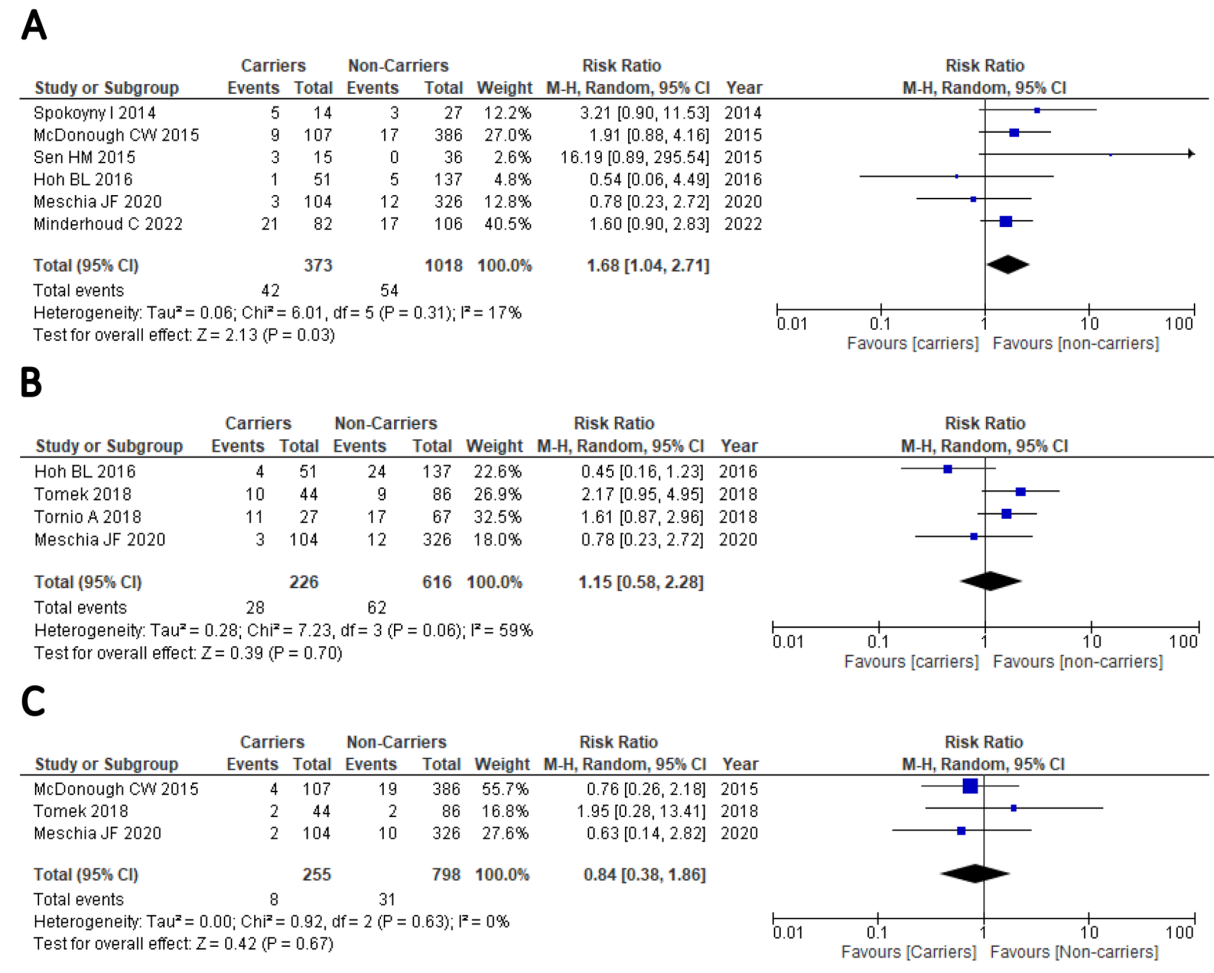
Forest plot for the comparison of carriers vs. non-carriers of CYP2C19 LOF alleles for the risk of stroke **(A)**, composite vascular events **(B)** or bleeding **(C)** among non-East Asian patients with stroke or TIA after receiving clopidogrel therapy

The summary results of the random-effect meta-analyses is shown in Table 2. In the overall analysis of 6 non-East Asian studies [17–19, 25, 28, 29], including a total of 1391 patients undergoing clopidogrel-based antiplatelet therapy, those carrying CYP2C19 LOF alleles were found at higher risk of stroke recurrence compared to non-carriers (RR: 1.68, 95%CI: 1.04–2.71, P = 0.03, Fig. 2A), and significant between-study heterogeneity was not observed (I^2^ = 17%, P = 0.31). The influence of each study on the overall effect-size estimate was evaluated by leave-one-out meta-analysis (Supplementary Fig. 1A), which showed that the pooled RR ranged from 1.53 (95%CI: 0.92–2.57, P = 0.10) when the study of Spokoyny et al. [17] was omitted, to 1.86 (95%CI: 1.16–2.97, P = 0.010) when the study of Meschia et al. [28] was excluded from the analysis, suggesting lack of robustness of the overall pooled result. Four studies enrolling a total of 842 patients receiving clopidogrel were available for the meta-analysis of the effect of carrying CYP2C19 LOF alleles on the risk of composite vascular events [19, 26–28]. No significant effect was observed for the comparison of carriers vs. non-carriers of CYP2C19 LOF alleles on the risk of composite vascular events (RR: 1.15, 95%CI: 0.58–2.28, P = 0.69, Fig. 2B), in presence of borderline not significant heterogeneity among studies (I^2^ = 59%, P = 0.06). Noteworthy, a significant impact of carrying CYP2C19 LOF alleles was found on the risk of composite vascular events when the study of Hoh et al. [19] was omitted from the meta-analysis (RR: 1.60, 95%CI: 1.01–2.53, P = 0.04, Supplementary Fig. 1B), suggesting again no robust estimation of the pooled effect size. Three studies [18, 27, 28], including a total of 1053 patients, were available for the meta-analysis of the effect of carrying CYP2C19 LOF alleles on bleeding risk. No association was detected in the pooled results (RR: 0.84, 95%CI: 0.38–1.86, P = 0.67, Fig. 2B) and no heterogeneity emerged between studies (I^2^ = 0%, P = 0.63). When carriers of 1 CYP2C19 LOF allele were analysed separately from carriers of 2 CYP2C19 LOF alleles, no significant results emerged in both comparisons (i.e. carriers of 1 CYP2C19 LOF allele vs. non-carriers and carriers of 2 CYP2C19 LOF alleles vs. non-carriers), as shown in Supplementary Figs. 1–3.

**Table 2:** Summary of random-effect meta-analyses for the association of CYP2C19 LOF alleles with clinical outcomes of non-East Asian patients with stroke or transient ischemic attack undergoing clopidogrel therapy

Outcome	Group (studies)	N° of carriers/ non-carriers	Test of association		Test of heterogeneity		Egger’s P-value
			RR (95%CI)	P-value	I^2^ (%)	P-value	
**Stroke**	All (n = 6)	373/1018	1.68 (1.04–2.71)	0.03	17	0.31	0.76
	EUR (n = 3)	92/275	2.69 (1.11–6.51)	0.03	0	0.38	
	Studies with at least 1 year of follow-up (n = 3)	173/559	1.96 (0.51–7.46)	0.32	42	0.18	
**Composite vascular events**	All (n = 4)	226/616	1.15 (0.58–2.28)	0.70	59	0.06	0.35
	EUR (n = 2)	71/153	1.63 (0.91–2.93)	0.10	0	0.85	
	Studies with at least 1 year of follow-up (n = 3)	122/290	1.23 (0.53–2.85)	0.62	69	0.04	
**Bleeding**	All (3)	255/798	0.84 (0.38–1.86)	0.67	0	0.63	0.53
	EUR (n = 2)	85/221	1.74 (0.58–5.23)	0.32	0	0.89	
	Studies with at least 1 year of follow-up (n = 2)	151/472	0.95 (0.37–2.39)	0.91	0	0.40	

CI, confidence interval; EUR, Europeans; I^2^, Higgins’ I-square test RR, risk ratio

### Publication Bias and Certainty of the Evidence Assessment

No statistical evidence of funnel plot asymmetry or small-study effects was detected in the pooled overall analyses for the three considered outcomes (Table 2), suggesting no publication bias in the results. The GRADEpro tool was used to rate the quality of the evidence on the association of CYP2C19 LOF allele status with the three clinical outcomes investigated. A moderate quality of evidence was observed for the risk of stroke, while a very low quality of evidence was rated for the risk of composite vascular events and bleeding (Supplementary Table 4).

### Subgroup and Sensitivity Analyses

**Figure 3:**
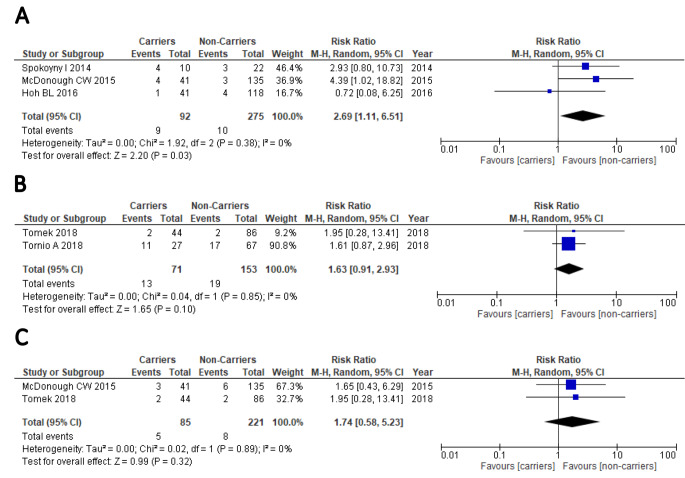
Forest plot for the comparison of carriers vs. non-carriers of CYP2C19 LOF alleles for the risk of stroke **(A)**, composite vascular events **(B)** or bleeding **(C)** in European ancestry patients with stroke or TIA after receiving clopidogrel therapy

Data for calculation of pooled clinical outcomes for the European subgroup (total stroke/TIA patients = 591) were available from five studies [17–19, 26, 27]. In the subgroup analysis conducted in patients with European ancestry (Fig. 3; Table 2), the pooled results showed that carriers of CYP2C19 LOF have an increased risk of stroke (RR: 2.69 (1.11–6.51, P = 0.03, Fig. 3A), but not of composite vascular events (Fig. 3B) or bleeding (Fig. 3C) after receiving clopidogrel-based antiplatelet therapy. Sensitivity analyses were also conducted based on studies with at least 1 year of patients’ follow-up [18, 19, 25–27]. Pooled results respectively for stroke (total stroke/TIA patients = 732), composite outcome events (total stroke/TIA patients = 412) and bleeding (total stroke/TIA patients = 623) showed no association for any of the three outcomes (Table 2).

## Discussion

Limited and conflicting data have been reported so far on the clinical utility of CYP2C19 genotyping in stroke/TIA patients of non-East Asian ancestries. The fact that 12 out of 15 studies included in the previous meta-analysis of Pan et al. [16] were conducted on East Asian patients, primarily of Han-Chinese ancestry, can be explained, among other reasons, by a higher frequency of CYP2C19 LOF alleles in this population in comparison to other ethnic groups, with intermediate and poor metabolizers accounting for about 59% of East Asians compared to 29% of white Europeans and 23% of Americans [12]. In addition, the incidence of stroke is higher in China than in USA and Europe [30, 31], with first-ever strokes per year in China accounting for almost a quarter of the global incident cases of stroke [32]. However, given population growth and ageing, the global lifetime risk of stroke in Western countries is expected to rise in the next few decades, with forecasts suggesting that the number of stroke patients will increase in Europe by 27% within 2047 [31] and that strokes events will double in the USA by 2050 [33]. Thus, given the recent publication of additional studies, in the present study we conducted an up-to-date meta-analysis to obtain a more precise estimation of the association between CYP2C19 LOF allele status and the clinical response to clopidogrel in non-East Asian patients with stroke or TIA.

The present updated meta-analysis comprises 8 studies with a total number of 1673 clopidogrel-treated stroke/TIA patients, that is more than twice as many non-East Asian patients compared with the previous meta-analysis of Pan et al. [16]. The pooled results showed that carriers of CYP2C19 LOF alleles are at significant higher risk of stroke after receiving clopidogrel-based antiplatelet therapy, compared to non-carriers (RR: 1.68, 95%CI: 1.04–2.71, P = 0.03), and this association was scored as “moderate quality” using the GRADEpro tool. In contrast, no difference was found in the risk of composite vascular events (RR: 1.15, 95%CI: 0.58–2.28) or bleeding (RR: 0.84, 95%CI: 0.38–1.86), despite a “very low quality” evidence was identified for these two outcomes. Similarly to the overall analysis, a significant association of CYP2C19 LOF alleles status was found with stroke in the subgroup of patients with European ancestry.

Overall, our findings provide moderate evidence that carriers of CYP2C19 LOF alleles derive less benefit from clopidogrel treatment after ischemic stroke or TIA, suggesting that CYP2C19 genetic testing may be considered to personalize antiplatelet therapy in non-East Asian patients. In such populations, if aspirin is contraindicated, clopidogrel dose escalation or antiplatelet agents not influenced by CYP2C19 genetic variants, such as ticagrelor and ticlopidine, could be alternative P2Y12 inhibitors for CYP2C19 LOF alleles carriers after ischemic stroke or TIA. It is noteworthy that our pooled estimates, which stem form data of non-East Asian studies, are in line with those obtained in the East Asian subgroup of stroke/TIA patients from the previous meta-analysis of Pan et al. [16], which found that East-Asian carriers of CYP2C19 LOF alleles are at higher risk of stroke in comparison with non-carriers (RR: 1.93, 95%CI 1.55–2.39, P < 0.00001), while no difference emerged among patients of East Asia in the risk of bleeding (RR: 0.92, 95%CI: 0.58–1.45, P = 0.72). Overall, these results suggest a similar impact of CYP2C19 LOF alleles on the efficacy and safety of clopidogrel in stroke/TIA patients of East-Asian ancestry compared to those of non-East Asian origin. Therefore, our findings further corroborate current CPIC and DPWG guidelines for CYP2C19 genotype-guided clopidogrel therapy in stroke/ TIA patients [12, 34], which provide evidence of a moderate strength recommendation of alternative antiplatelet agents for CYP2C19 LOF allele carriers and do not suggest specific recommendations according to patient’s ethnicity, although based primarily on evidence available in Asian patients.

We acknowledge several limitations of our study that should be taken into account for the correct interpretation of the results. Firstly, the present systematic review consists of a limited number of studies with a relatively low sample size. Despite our attempt to conduct a comprehensive pooled analysis of all available published reports, some publications were not eligible for the systematic review because of unavailability of corresponding authors to provide the data requested for the effect size calculation. Therefore, great caution is needed in the interpretation of our pooled risk estimates, a consideration which is also suggested by the results of leave-one-out meta-analyses showing lack of robustness of pooled estimates. Secondly, according to the inclusion criteria of the present systematic review, we considered only non-East Asian studies enrolling patients with stroke or TIA undergoing clopidogrel-based antiplatelet therapy. However, very few patients of East Asian ancestry could have been recruited in some of the included studies, as shown in Supplementary Table 2. Nevertheless, a significant association was still found in the subgroup of patients with European ancestry between carriers of CYP2C19 LOF alleles and higher risk of stroke, after clopidogrel-based antiplatelet therapy. Thirdly, the studies of the present systematic review differ with respect to some characteristics such as clopidogrel-based regimen, follow-up duration and CYP2C19 variant alleles analysed, which may have an impact on the effect size estimation. Nevertheless, little or no heterogeneity was observed in the pooled results, as shown by the I^2^ statistics. Fourthly, in almost all the included studies, investigation of loss-of-function CYP2C19 alleles was limited to CYP2C19*2 and CYP2C19*3 alleles, which represent the most frequent CYP2C19 LOF alleles among all populations. Other CYP2C19 alleles such as CYP2C19*9 and CYP2C19*10, which are known to determine a reduced enzymatic activity [12], have been overlooked by the authors of the studies included in the present systematic review. However, the risk that this could have affected our findings is rather low, given the very low frequency of CYP2C19*9 and CYP2C19*10 alleles among the different populations [12]. Moreover, our pooled estimates are not adjusted by factors known to have an impact on metabolic activation of clopidogrel via CYP2C19. For instance, the co-administration of CYP2C19 inhibitors, such as proton pump inhibitors (PPIs) or some antidepressant drugs, reduces the efficacy of clopidogrel on platelets and may thus worsen the clinical outcome [35], while cigarette smoking is known to enhance clopidogrel-induced platelet inhibition, which may explain the higher relative benefit of clopidogrel among smokers [36, 37]. To address the latter limitation, a meta-analysis of individual participant data should be performed to obtain adjusted RRs and to assess the impact of factors, such as PPIs or antidepressants coadministration as well as cigarette smoking, on the association of CYP2C19 genotype with efficacy and safety of clopidogrel therapy in stroke/TIA patients. On the other hand, clinical risk factors and the CYP2C19 genotype can be integrated by the ABCD-GENE score to estimate the efficacy of clopidogrel-aspirin therapy [38]. The ABCD-GENE score, which incorporates age, BMI, chronic kidney disease, diabetes mellitus and CYP2C19 loss-of-function alleles, has been demonstrated among Chinese minor stroke/TIA patients to identify subjects at increased risk of stroke recurrence following clopidogrel-aspirin therapy [39]. Future investigation is thus required to assess validity of the ABCD-GENE score tool in stroke/TIA patients of other ethnic origins, including Europeans. Furthermore, it is well known that CYP2C19*2 and *3 alleles are associated to a decreased antiplatelet activity, as demonstrated by ex vivo platelet reactivity testing in on-treatment patients [15, 40, 41]. However, platelet reactivity analysis was conducted by only one of the included studies [27], which results were consistent with a strong association of platelet activity with CYP2C19*2 allele status. Lastly, only two studies [27, 29] included in our analysis have reported the ischemic stroke subtypes according to TOAST classification and, in both these studies, cases with undetermined cause were a large proportion (40% and 53.7%, respectively). This could have impacted the strength of the association between CYP2C19 LOF allele status and efficacy of clopidogrel therapy in preventing stroke recurrence, given that in a proportion of these patients antiplatelet therapy might not have been the best secondary prevention strategy. In fact, among other causes, occult non-valvular atrial fibrillation (AF) could have been the culprit of some cases of stroke recurrence, which therefore should not be considered linked to the lack of clopidogrel efficacy in LOF allele carriers. Moreover, concomitant presence of known and properly treated AF in those rare patients requiring also an antiplatelet therapy for several reasons could be potential source of stroke recurrence, given the possibility of anticoagulation failure or lack of patients’ compliance. Unfortunately, only [28] and [29] have reported the number of patients with known AF, which were a small number (Supplementary Table 2). Nevertheless, the pharmacogenetic analysis of the ACTIVE trial [42] has shown a lack of difference between CYP2C19 genotypes and outcomes in AF patients.

In conclusion, the present systematic review provides updated pooled risk estimates for the association of CYP2C19 LOF allele status with efficacy and safety of clopidogrel-based antiplatelet therapy in non-East Asian patients with stroke or TIA, including Europeans. The results of pooled analyses from non-East Asian studies support that carriers of CYP2C19 LOF alleles have a significant increased risk of stroke following TIA or ischemic stroke when treated with clopidogrel. These results provide some evidence of potential clinical utility of CYP2C19 genotyping to personalize antiplatelet therapy in non-East Asian patients with stroke/TIA, including those of European ancestry. However, due to the limited sample size of this systematic review, further large and possibly multicentric studies enrolling stroke/TIA patients of European and other non-East Asian ancestry are warranted. Such studies are strongly needed not only to confirm our findings but also to investigate gene–gene and gene–environment interactions, along with the adjustment of confounding factors, for the association of CYP2C19 genotype with efficacy and safety of clopidogrel in patients with stroke or TIA.

## Electronic Supplementary Material

Below is the link to the electronic supplementary material.

Supplementary Material 1

## Data Availability

The original contributions presented in the study were included in the article/Supplementary Material. Further inquiries can be directed to the corresponding authors.

## References

[1] GBD 2019 Stroke Collaborators. Global, regional, and national burden of stroke and its risk factors, 1990–2019: a systematic analysis for the Global Burden of Disease Study 2019. Lancet Neurol. 2021; 20:795–820. 10.1016/S1474-4422(21)00252-0 PMC8443449 34487721

[2] Feigin VL, Brainin M, Norrving B, et al. World Stroke Organization (WSO): global Stroke fact sheet 2022. Int J Stroke. 2022;17:18–29. 34986727 10.1177/17474930211065917

[3] Donkor ES. Stroke in the 21st Century: a snapshot of the Burden, Epidemiology, and Quality of Life. Stroke Res Treat. 2018;3238165. 10.1155/2018/3238165 PMC6288566 30598741

[4] Easton JD, Saver JL, Albers GW, healthcare professionals from the American Heart Association/American Stroke Association Stroke Council. Definition and evaluation of transient ischemic attack: a scientific statement for ; Council on Cardiovascular Surgery and Anesthesia; Council on Cardiovascular Radiology and Intervention; Council on Cardiovascular Nursing; and the Interdisciplinary Council on Peripheral Vascular Disease. The American Academy of Neurology affirms the value of this statement as an educational tool for neurologists. Stroke. 2009; 40:2276–93. 10.1161/STROKEAHA.108.192218 19423857

[5] Rothwell PM, Warlow CP. Timing of TIAs preceding Stroke: time window for prevention is very short. Neurology. 2005;64:817–20. 15753415 10.1212/01.WNL.0000152985.32732.EE

[6] Kleindorfer DO, Towfighi A, Chaturvedi S, et al. 2021 Guideline for the Prevention of Stroke in patients with Stroke and transient ischemic Attack: a Guideline from the American Heart Association/American Stroke Association. Stroke. 2021;52:e364–e467. 34024117 10.1161/STR.0000000000000375

[7] Dawson J, Béjot Y, Christensen LM, et al. European Stroke Organisation (ESO) guideline on pharmacological interventions for long-term secondary prevention after ischaemic Stroke or transient ischaemic Attack. Eur Stroke J. 2022;7:I–II. 36082250 10.1177/23969873221100032 PMC9446324

[8] Gladstone DJ, Lindsay MP, Douketis J, et al. Canadian Stroke best practice recommendations: secondary Prevention of Stroke Update 2020. Can J Neurol Sci. 2022;49:315–37. 34140063 10.1017/cjn.2021.127

[9] John S, Katzan I. Recurrent Stroke while on Antiplatelet Therapy. Neurol Clin. 2015;33:475–89. 25907917 10.1016/j.ncl.2014.12.007

[10] Stahmeyer JT, Stubenrauch S, Geyer S, Weissenborn K, Eberhard S. The frequency and timing of recurrent Stroke: An Analysis of Routine Health Insurance Data. Dtsch Arztebl Int. 2019;116:711–17. 31711561 10.3238/arztebl.2019.0711 PMC6891883

[11] Shah J, Liu S, Yu W. Contemporary antiplatelet therapy for secondary Stroke prevention: a narrative review of current literature and guidelines. Stroke Vasc Neurol. 2022;7:406–14. 35393359 10.1136/svn-2021-001166 PMC9614124

[12] Lee CR, Luzum JA, Sangkuhl K, et al. Clinical pharmacogenetics implementation Consortium Guideline for CYP2C19 genotype and clopidogrel therapy: 2022 update. Clin Pharmacol Ther. 2022;112:959–67. 35034351 10.1002/cpt.2526 PMC9287492

[13] Sangkuhl K, Klein TE, Altman RB. Clopidogrel pathway. Pharmacogenet Genomics. 2010;20:463–5. 20440227 10.1097/FPC.0b013e3283385420 PMC3086847

[14] Ford NF. The metabolism of Clopidogrel: CYP2C19 is a minor pathway. J Clin Pharmacol. 2016;56:1474–83. 27196064 10.1002/jcph.769

[15] Kim KA, Park PW, Hong SJ, Park JY. The effect of CYP2C19 polymorphism on the pharmacokinetics and pharmacodynamics of clopidogrel: a possible mechanism for clopidogrel resistance. Clin Pharmacol Ther. 2008;84:236–42. 18323861 10.1038/clpt.2008.20

[16] Pan Y, Chen W, Xu Y, et al. Genetic polymorphisms and Clopidogrel Efficacy for Acute ischemic Stroke or transient ischemic Attack: a systematic review and Meta-analysis. Circulation. 2017;135:21–33. 27806998 10.1161/CIRCULATIONAHA.116.024913

[17] Spokoyny I, Barazangi N, Jaramillo V, et al. Reduced clopidogrel metabolism in a multiethnic population: prevalence and rates of recurrent cerebrovascular events. J Stroke Cerebrovasc Dis. 2014;23:694–8. 23849748 10.1016/j.jstrokecerebrovasdis.2013.06.008

[18] McDonough CW, McClure LA, Mitchell BD, et al. CYP2C19 metabolizer status and clopidogrel efficacy in the secondary Prevention of small subcortical strokes (SPS3) study. J Am Heart Assoc. 2015;4:e001652. 26019129 10.1161/JAHA.114.001652 PMC4599525

[19] Hoh BL, Gong Y, McDonough CW, et al. CYP2C19 and CES1 polymorphisms and efficacy of clopidogrel and aspirin dual antiplatelet therapy in patients with symptomatic intracranial atherosclerotic Disease. J Neurosurg. 2016;124:1746–51. 26587656 10.3171/2015.6.JNS15795 PMC4915569

[20] Yan Y, Hao R, Zhao X et al. Relationship between CYP2C19*2, *3 gene polymorphism and the recurrence in ischemic Stroke patients treated with clopidogrel in China: a meta-analysis. J Stroke Cerebrovasc 2022; Dis 31(11):106798. 10.1016/j.jstrokecerebrovasdis.2022.106798 36215918

[21] McDermott JH, Leach M, Sen D, et al. The role of CYP2C19 genotyping to guide antiplatelet therapy following ischemic Stroke or transient ischemic Attack. Expert Rev Clin Pharmacol. 2022;15:811–25. 35912831 10.1080/17512433.2022.2108401 PMC9612933

[22] Page MJ, McKenzie JE, Bossuyt PM, et al. The PRISMA 2020 statement: an updated guideline for reporting systematic reviews. BMJ. 2021;372:n71. 33782057 10.1136/bmj.n71 PMC8005924

[23] Adams HP Jr, Bendixen BH, Kappelle LJ, et al. Classification of subtype of acute ischemic Stroke. Definitions for use in a multicenter clinical trial. TOAST. Trial of Org 10172 in Acute Stroke Treatment. Stroke. 1993;24:35–41. 7678184 10.1161/01.str.24.1.35

[24] Borenstein M, Hedges LV, Higgins JP, Rothstein HR. A basic introduction to fixed-effect and random-effects models for meta-analysis. Res Synth Methods. 2010;1:97–111. 26061376 10.1002/jrsm.12

[25] Sen HM, Silan F, Silan C, Degirmenci Y, Ozisik Kamaran HI. Effects of CYP2C19 and P2Y12 gene polymorphisms on clinical results of patients using clopidogrel after Acute Ischemic Cerebrovascular Disease. Balkan J Med Genet. 2015;17:37–41. 25937796 10.2478/bjmg-2014-0072 PMC4413440

[26] Tornio A, Flynn R, Morant S, et al. Investigating Real-World Clopidogrel Pharmacogenetics in Stroke using a Bioresource Linked to Electronic Medical records. Clin Pharmacol Ther. 2018;103:281–6. 28653333 10.1002/cpt.780 PMC5813097

[27] Tomek A, Matʼoška V, Frýdmanová A, et al. Impact of CYP2C19 polymorphisms on clinical outcomes and antiplatelet potency of Clopidogrel in caucasian poststroke survivors. Am J Ther. 2018;25:e202–12. 29509167 10.1097/MJT.0000000000000416

[28] Meschia JF, Walton RL, Farrugia LP, et al. Efficacy of Clopidogrel for Prevention of Stroke based on CYP2C19 allele Status in the POINT trial. Stroke. 2020;51:2058–65. 32568642 10.1161/STROKEAHA.119.028713 PMC9523583

[29] Minderhoud C, Otten LS, Hilkens PHE, van den Broek MPH, Harmsze AM. Increased frequency of CYP2C19 loss-of-function alleles in clopidogrel-treated patients with recurrent cerebral ischemia. Br J Clin Pharmacol. 2022;88:3335–40. 35176816 10.1111/bcp.15282

[30] Virani SS, Alonso A, Benjamin EJ, et al. Heart Disease and Stroke Statistics-2020 update: a Report from the American Heart Association. Circulation. 2020;141(9):e139–e596. 31992061 10.1161/CIR.0000000000000757

[31] Wafa HA, Wolfe CDA, Emmett E, et al. Burden of Stroke in Europe: thirty-year projections of incidence, prevalence, deaths, and disability-adjusted life years. Stroke. 2020;51:2418–27. 32646325 10.1161/STROKEAHA.120.029606 PMC7382540

[32] GBD 2019 Diseases and Injuries Collaborators. Global burden of 369 Diseases and injuries in 204 countries and territories, 1990–2019: a systematic analysis for the global burden of Disease Study 2019. Lancet. 2020;396:1204–22. 33069326 10.1016/S0140-6736(20)30925-9 PMC7567026

[33] Gorelick PB. The future of Stroke prevention by risk factor modification. Handb Clin Neurol. 2009;94:1261–76. 18793900 10.1016/S0072-9752(08)94063-X

[34] Royal Dutch Pharmacists Association (KNMP). Dutch Pharmacogenetics Working Group (DPWG). Pharmacogenetic Guidelines, Netherlands. Clopidogrel – CYP2C19, https://www.knmp.nl/dossiers/farmacogenetica (Accessed 1st August 2023).

[35] Anderson CD, Biffi A, Greenberg SM, Rosand J. Personalized approaches to clopidogrel therapy: are we there yet? Stroke. 2010;41:2997–3002. 21030701 10.1161/STROKEAHA.110.594069 PMC3014683

[36] Bliden KP, Baker BA, Nolin TD, et al. Thienopyridine efficacy and cigarette Smoking status. Am Heart J. 2013;165:693–703. 23622905 10.1016/j.ahj.2012.12.024

[37] Zhao ZG, Chen M, Peng Y, et al. The impact of Smoking on clinical efficacy and pharmacodynamic effects of clopidogrel: a systematic review and meta-analysis. Heart. 2014;100:192–9. 23749792 10.1136/heartjnl-2013-304138

[38] Angiolillo DJ, Capodanno D, Danchin N, et al. Derivation, validation, and Prognostic Utility of a prediction rule for nonresponse to Clopidogrel: the ABCD-GENE score. JACC Cardiovasc Interv. 2020;13:606–17. 32139218 10.1016/j.jcin.2020.01.226

[39] Dai L, Xu J, Yan H, et al. Application of Age, Body Mass Index, chronic Kidney Disease, Diabetes, and genotyping score for efficacy of Clopidogrel: secondary analysis of the CHANCE trial. Stroke. 2022;53:465–72. 34666508 10.1161/STROKEAHA.120.033049

[40] Shuldiner AR, O’Connell JR, Bliden KP, et al. Association of cytochrome P450 2C19 genotype with the antiplatelet effect and clinical efficacy of clopidogrel therapy. JAMA. 2009;302:849–57. 19706858 10.1001/jama.2009.1232 PMC3641569

[41] Jeong YH, Tantry US, Kim IS, et al. Effect of CYP2C19*2 and *3 loss-of-function alleles on platelet reactivity and adverse clinical events in east Asian acute Myocardial Infarction survivors treated with clopidogrel and aspirin. Circ Cardiovasc Interv. 2011;4:585–94. 22045970 10.1161/CIRCINTERVENTIONS.111.962555

[42] Paré G, Mehta SR, Yusuf S, et al. Effects of CYP2C19 genotype on outcomes of clopidogrel treatment. N Engl J Med. 2010;363:1704–14. 20979470 10.1056/NEJMoa1008410

