# Supplementary material for: Impact of CYP2C19 Genotype on Efficacy and Safety of Clopidogrel-based Antiplatelet Therapy in Stroke or Transient Ischemic Attack Patients: An Updated Systematic Review and Meta-analysis of Non-East Asian Studies
Source: Cardiovasc Drugs Ther. 2023 Dec 1;38(6):1397–407. doi: 10.1007/s10557-023-07534-0 (PMC11680632; doi:10.1007/s10557-023-07534-0)
Supplement: Supplementary file 1 — Supplementary Material 1 [file 10557_2023_7534_MOESM1_ESM.pdf]

Supplementary Fig. 1

Results of leave-one-out sensitivity meta-analyses for the effect of carrying CYP2C19 LOF alleles on the risk of stroke (A), composite vascular events (B) or bleeding (C) in non-East Asian patients with stroke or TIA after receiving clopidogrel-based antiplatelet therapy. ES, effect size, i.e. risk ratio; CI, confidence interval; N, total sample; Sig., statistical significance.

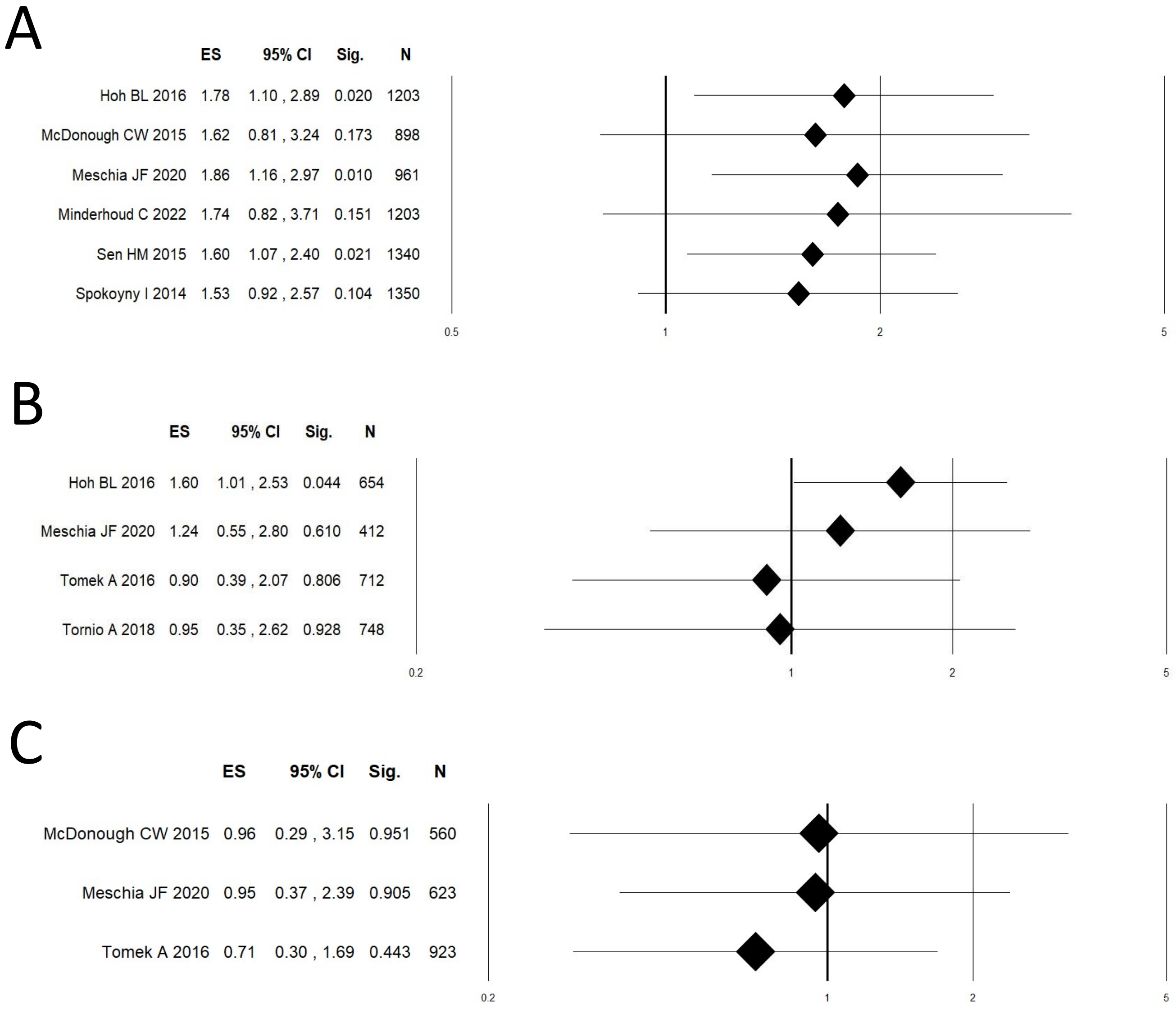

Supplementary Fig. 2

Forest plot for the comparison of carriers of 1 CYP2C19 loss-of-function (LOF) allele vs non-carriers for the risk of stroke (A), composite vascular events (B) or bleeding (C) among non-East Asian patients with stroke or TIA after receiving clopidogrel therapy.

A

	1 CYP2C19 LOF Carriers		Non-Carriers			Risk Ratio	
Study or Subgroup	Events	Total	Events	Total	Weight	M-H, Random, 95% CI	Year
Spokoyny I 2014	4	12	3	27	17.5%	3.00 [0.79, 11.38]	2014
Sen HM 2015	2	13	0	36	4.0%	13.21 [0.68, 258.46]	2015
Meschia JF 2020	3	93	12	326	19.7%	0.88 [0.25, 3.04]	2020
Minderhoud C 2022	20	72	17	106	58.9%	1.73 [0.98, 3.07]	2022
**Total (95% CI)**		**190**		**495**	**100.0%**	**1.81 [0.99, 3.29]**	
Total events	29		32				

Heterogeneity: Tau² = 0.07; Chi² = 3.59, df = 3 (P = 0.31); I² = 16%
Test for overall effect: Z = 1.93 (P = 0.05)

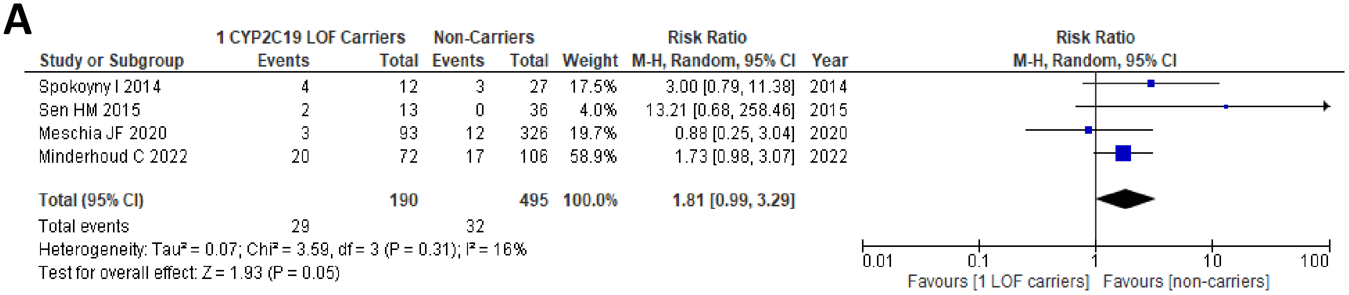

B

	1 CYP2C19 LOF Carriers		Non-Carriers			Risk Ratio	
Study or Subgroup	Events	Total	Events	Total	Weight	M-H, Random, 95% CI	Year
Tomek 2018	10	44	9	86	63.5%	2.17 [0.95, 4.95]	2018
Meschia JF 2020	3	93	12	326	36.5%	0.88 [0.25, 3.04]	2020
**Total (95% CI)**		**137**		**412**	**100.0%**	**1.56 [0.66, 3.69]**	
Total events	13		21				

Heterogeneity: Tau² = 0.13; Chi² = 1.44, df = 1 (P = 0.23); I² = 31%
Test for overall effect: Z = 1.01 (P = 0.31)

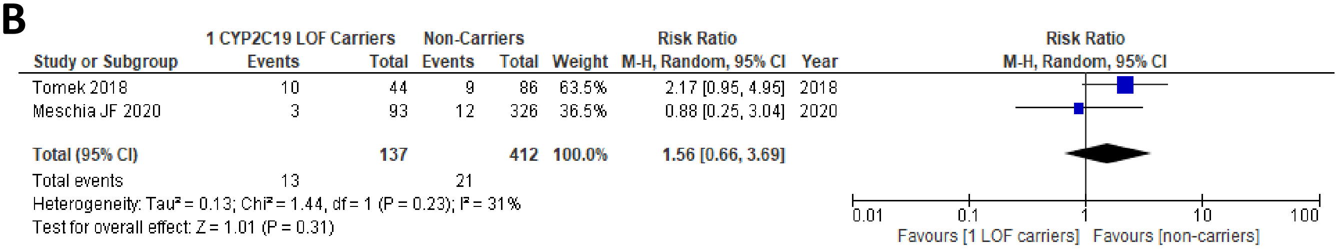

C

	1 CYP2C19 LOF Carriers		Non-Carriers			Risk Ratio	
Study or Subgroup	Events	Total	Events	Total	Weight	M-H, Random, 95% CI	Year
Tomek 2018	2	44	6	86	48.1%	0.65 [0.14, 3.10]	2018
Meschia JF 2020	2	93	10	326	51.9%	0.70 [0.16, 3.14]	2020
**Total (95% CI)**		**137**		**412**	**100.0%**	**0.68 [0.23, 1.99]**	
Total events	4		16				

Heterogeneity: Tau² = 0.00; Chi² = 0.00, df = 1 (P = 0.95); I² = 0%
Test for overall effect: Z = 0.71 (P = 0.48)

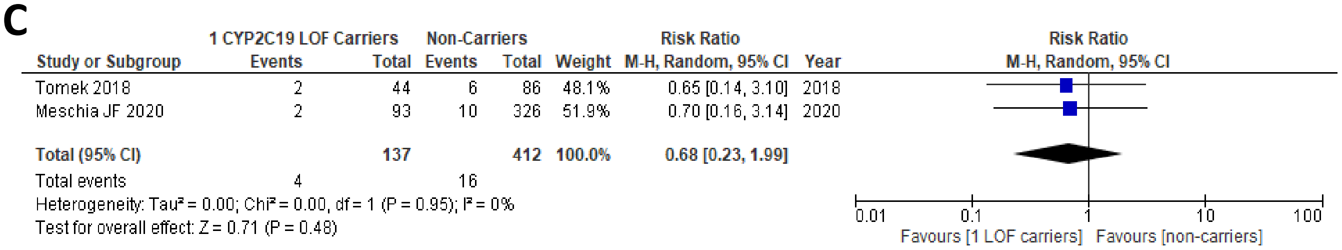

Supplementary Fig. 3

Forest plot for the comparison of carriers of 2 CYP2C19 loss-of-function (LOF) alleles vs non-carriers for the risk of stroke (A), composite vascular events (B) or bleeding (C) among non-East Asian patients with stroke or TIA after receiving clopidogrel therapy.

A

	2 CYP2C19 LOF carriers		Non-Carriers			Risk Ratio	
**Study or Subgroup**	**Events**	**Total**	**Events**	**Total**	**Weight**	**M-H, Random, 95% CI**	**Year**
Spokoyny I 2014	1	2	3	27	31.1%	4.50 [0.78, 25.87]	2014
Sen HM 2015	1	2	0	36	19.1%	37.00 [1.89, 725.91]	2015
Meschia JF 2020	0	11	8	178	20.6%	0.88 [0.05, 14.31]	2020
Minderhoud C 2022	1	10	17	106	29.2%	0.62 [0.09, 4.21]	2022
**Total (95% CI)**		**25**		**347**	**100.0%**	**2.70 [0.50, 14.69]**	
Total events	3		28				

Heterogeneity: Tau² = 1.61; Chi² = 6.65, df = 3 (P = 0.08); I² = 55%
Test for overall effect: Z = 1.15 (P = 0.25)

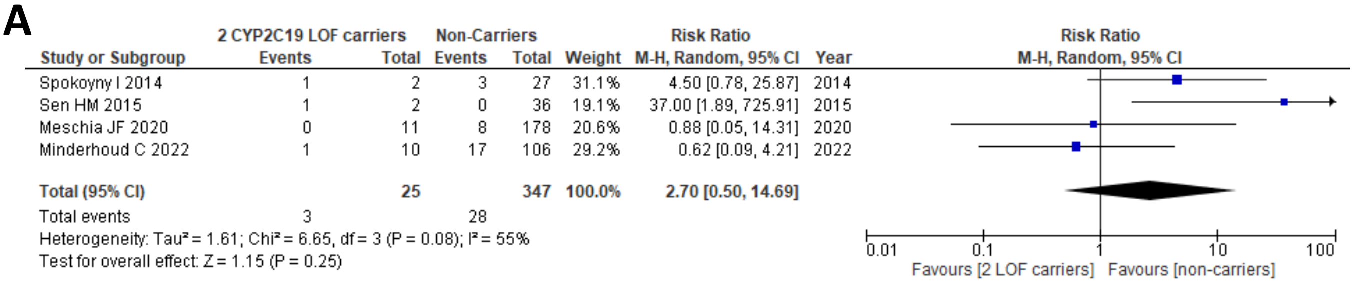

B

	2 CYP2C19 LOF Carriers		Non-Carriers			Risk Ratio	
**Study or Subgroup**	**Events**	**Total**	**Events**	**Total**	**Weight**	**M-H, Random, 95% CI**	**Year**
Tomek 2018	3	12	9	86	85.1%	2.39 [0.75, 7.61]	2018
Meschia JF 2020	0	11	12	326	14.9%	1.09 [0.07, 17.35]	2020
**Total (95% CI)**		**23**		**412**	**100.0%**	**2.13 [0.73, 6.19]**	
Total events	3		21				

Heterogeneity: Tau² = 0.00; Chi² = 0.27, df = 1 (P = 0.60); I² = 0%
Test for overall effect: Z = 1.38 (P = 0.17)

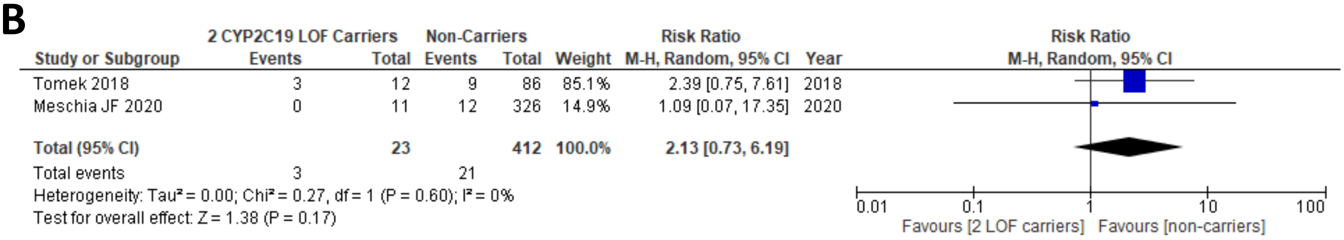

C

	2 CYP2C19 LOF Carriers		Non-Carriers			Risk Ratio	
**Study or Subgroup**	**Events**	**Total**	**Events**	**Total**	**Weight**	**M-H, Random, 95% CI**	**Year**
Tomek 2018	0	12	6	86	49.3%	0.51 [0.03, 8.61]	2018
Meschia JF 2020	0	11	10	326	50.7%	1.30 [0.08, 20.87]	2020
**Total (95% CI)**		**23**		**412**	**100.0%**	**0.82 [0.11, 5.95]**	
Total events	0		16				

Heterogeneity: Tau² = 0.00; Chi² = 0.22, df = 1 (P = 0.64); I² = 0%
Test for overall effect: Z = 0.19 (P = 0.85)

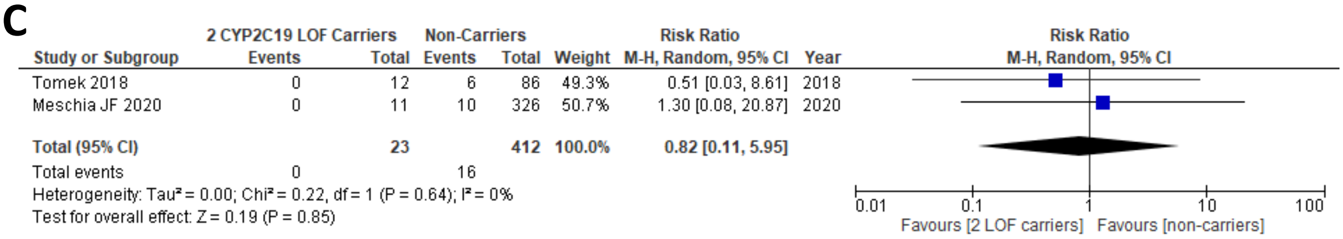

Supplementary Table 1. PRISMA 2020 Checklist.

Section and Topic	Item #	Checklist item	Reported in the article section entitled (on page #)
**TITLE**			
Title	1	Identify the report as a systematic review.	Title (1)
**ABSTRACT**			
Abstract	2	See the PRISMA 2020 for Abstracts checklist.	Abstract (2)
**INTRODUCTION**			
Rationale	3	Describe the rationale for the review in the context of existing knowledge.	Introduction (3-4)
Objectives	4	Provide an explicit statement of the objective(s) or question(s) the review addresses.	Introduction (4)
**METHODS**			
Eligibility criteria	5	Specify the inclusion and exclusion criteria for the review and how studies were grouped for the syntheses.	Search and inclusion/exclusion criteria (5)
Information sources	6	Specify all databases, registers, websites, organisations, reference lists and other sources searched or consulted to identify studies. Specify the date when each source was last searched or consulted.	Search and inclusion/exclusion criteria (5)
Search strategy	7	Present the full search strategies for all databases, registers and websites, including any filters and limits used.	Search and inclusion/exclusion criteria (5)
Selection process	8	Specify the methods used to decide whether a study met the inclusion criteria of the review, including how many reviewers screened each record and each report retrieved, whether they worked independently, and if applicable, details of automation tools used in the process.	Search and inclusion/exclusion criteria (5-6)
Data collection process	9	Specify the methods used to collect data from reports, including how many reviewers collected data from each report, whether they worked independently, any processes for obtaining or confirming data from study investigators, and if applicable, details of automation tools used in the process.	Data extraction (6)
Data items	10a	List and define all outcomes for which data were sought. Specify whether all results that were compatible with each outcome domain in each study were sought (e.g. for all measures, time points, analyses), and if not, the methods used to decide which results to collect.	Data extraction (6)
	10b	List and define all other variables for which data were sought (e.g. participant and intervention characteristics, funding sources). Describe any assumptions made about any missing or unclear information.	Data extraction (6)
Study risk of bias assessment	11	Specify the methods used to assess risk of bias in the included studies, including details of the tool(s) used, how many reviewers assessed each study and whether they worked independently, and if applicable, details of automation tools used in the process.	Assessment of study quality and quality of evidence (6-7)
Effect measures	12	Specify for each outcome the effect measure(s) (e.g. risk ratio, mean difference) used in the synthesis or presentation of results.	Statistical analysis (7)
Synthesis methods	13a	Describe the processes used to decide which studies were eligible for each synthesis (e.g. tabulating the study intervention characteristics and comparing against the planned groups for each synthesis (item #5)).	N/A
	13b	Describe any methods required to prepare the data for presentation or synthesis, such as handling of missing summary statistics, or data conversions.	N/A

Section and Topic	Item #	Checklist item	Reported in the article section entitled (on page #)
	13c	Describe any methods used to tabulate or visually display results of individual studies and syntheses.	Statistical analysis (7)
	13d	Describe any methods used to synthesize results and provide a rationale for the choice(s). If meta-analysis was performed, describe the model(s), method(s) to identify the presence and extent of statistical heterogeneity, and software package(s) used.	Statistical analysis (7)
	13e	Describe any methods used to explore possible causes of heterogeneity among study results (e.g. subgroup analysis, meta-regression).	Statistical analysis (7)
	13f	Describe any sensitivity analyses conducted to assess robustness of the synthesized results.	Statistical analysis (7)
Reporting bias assessment	14	Describe any methods used to assess risk of bias due to missing results in a synthesis (arising from reporting biases).	Statistical analysis (7-8)
Certainty assessment	15	Describe any methods used to assess certainty (or confidence) in the body of evidence for an outcome.	Assessment of study quality and quality of evidence (7)
**RESULTS**			
Study selection	16a	Describe the results of the search and selection process, from the number of records identified in the search to the number of studies included in the review, ideally using a flow diagram.	General characteristics and quality of included studies (9), Fig. 1
	16b	Cite studies that might appear to meet the inclusion criteria, but which were excluded, and explain why they were excluded.	Fig. 1
Study characteristics	17	Cite each included study and present its characteristics.	General characteristics and quality of included studies (9), Table 1, Suppl. Table 2
Risk of bias in studies	18	Present assessments of risk of bias for each included study.	General characteristics and quality of included studies (9), Table 1, Suppl.Table 3
Results of individual studies	19	For all outcomes, present, for each study: (a) summary statistics for each group (where appropriate) and (b) an effect estimate and its precision (e.g. confidence/credible interval), ideally using structured tables or plots.	Fig. 2, Fig. 3, Suppl. Fig. 1, Suppl. Fig. 2, Suppl. Fig. 3, Table 2
Results of syntheses	20a	For each synthesis, briefly summarise the characteristics and risk of bias among contributing studies.	Quantitative data synthesis (10)
	20b	Present results of all statistical syntheses conducted. If meta-analysis was done, present for each the summary estimate and its precision (e.g. confidence/credible interval) and measures of statistical heterogeneity. If comparing groups, describe the direction of the effect.	Quantitative data synthesis (10), Fig. 2, Fig. 3, Table 2, Suppl. Fig. 1, Suppl. Fig. 2, Suppl. Fig. 3
	20c	Present results of all investigations of possible causes of heterogeneity among study results.	Subgroup and sensitivity analyses (11), Fig. 3, Table 2
	20d	Present results of all sensitivity analyses conducted to assess the robustness of the synthesized results.	Quantitative data synthesis (10), Subgroup and sensitivity analyses (11), Table 2, Suppl. Fig. 1
Reporting biases	21	Present assessments of risk of bias due to missing results (arising from reporting biases) for each synthesis assessed.	Quantitative data synthesis

Section and Topic	Item #	Checklist item	Reported in the article section entitled (on page #)
			(10), Table 2, publication bias and certainty of the evidence assessment (11)
Certainty of evidence	22	Present assessments of certainty (or confidence) in the body of evidence for each outcome assessed.	Publication bias and certainty of the evidence assessment (11), Suppl. Table 4
**DISCUSSION**			
Discussion	23a	Provide a general interpretation of the results in the context of other evidence.	Discussion (12, 13)
	23b	Discuss any limitations of the evidence included in the review.	Discussion (13-15)
	23c	Discuss any limitations of the review processes used.	Discussion (13-15)
	23d	Discuss implications of the results for practice, policy, and future research.	Discussion (15)
**OTHER INFORMATION**			
Registration and protocol	24a	Provide registration information for the review, including register name and registration number, or state that the review was not registered.	Methods, Search and inclusion/exclusion criteria (4)
	24b	Indicate where the review protocol can be accessed, or state that a protocol was not prepared.	Methods, Search and inclusion/exclusion criteria (4)
	24c	Describe and explain any amendments to information provided at registration or in the protocol.	N/A
Support	25	Describe sources of financial or non-financial support for the review, and the role of the funders or sponsors in the review.	Author Declarations, Funding (24)
Competing interests	26	Declare any competing interests of review authors.	Author Declarations, Competing interests (24)
Availability of data, code and other materials	27	Report which of the following are publicly available and where they can be found: template data collection forms; data extracted from included studies; data used for all analyses; analytic code; any other materials used in the review.	Author Declarations, Availability of data and material (24)

From: Page MJ, McKenzie JE, Bossuyt PM, Boutron I, Hoffmann TC, Mulrow CD, et al. The PRISMA 2020 statement: an updated guideline for reporting systematic reviews. BMJ 2021;372:n71. doi: 10.1136/bmj.n71

For more information, visit: http://www.prisma-statement.org/

Supplementary Table 2. Additional characteristics of studies included in the systematic review.

First author [Ref] (year)	East Asian patients, N (%)	Atrial Fibrillation, N (%)	Other concomitant antithrombotics, N (%)	Concomitant drugs		Smokers, N (%)
				PPIs, N (%)	Antidepressants, N (%)	
Spokoyny I et al. [17] (2014)	NR (<11.0)*	NR	NR	9 (20.9)	NR	6 (14.0)
Sen HM et al. [25] (2015)	0 (0.0)	NR	NR	NR	NR	NR
McDonough CW et al. [18] (2015)	0 (0.0)	NR	NR	NR	NR	NR
Hoh BL et al. [19] (2016)	NR (<2.7)*	NR	NR	109 (58.0)	NR	109 (58.0)
Tornio A et al. [26] (2018)	NR (<0.3)*	NR	NR	NR	NR	NR
Tomek A et al. [27] (2018)	0 (0.0)	NR	0 (0.0)	27 (20.8)	NR	NR
Meschia JF et al. [28] (2020)	NR (<6.1)*	7 (1.5)	NR	NR	NR	97 (21.2)
Minderhoud C et al. [29] (2022)	0 (0.0)	15 (8.0)	aspirin: 17 (9.0)<br>other anticoagulant: 4 (2.1)	93 (48.5)	NR	38 (20.2)

*In this study, the number of East- Asian patients was not specified, but it was less than the reported percentage, which also include other ethnicities (e.g. Blacks).

Abbreviations: NR, not reported; PPI, proton pump inhibitor.

Supplementary Table 3. Assessment of methodological study quality by using the Newcastle-Ottawa Scale.

Study (year of pubblication)	***Selection***				***Comparability***		***Outcome***			***Total score***
	Representativeness of the exposed cohort	*Selection of the non-exposed cohort*	*Ascertainment of exposure*	*Demonstration that outcome of interest was not present at start of study*	*Comparability of cohorts on the basis of the design or analysis*		*Assessment of outcome*	*Was follow-up long enough for outcomes to occur*	*Adequacy of follow up of cohorts*	
					Age	*Any other additional factor*				
Spokoyny I et al. [17] (2014)	*	*	*	*						4
Sen HM et al. [25] (2015)	*	*	*	*				*	*	6
McDonough CW et al. [18] (2015)	*	*	*	*	*	*	*	*	*	9
Hoh BL et al. [19] (2016)	*	*	*	*	*	*		*	*	8
Tornio A et al. [26] (2018)	*	*	*	*	*	*	*	*	*	9
Tomek A et al. [27] (2018)	*	*	*	*	*	*		*	*	8
Meschia JF et al. [28] (2020)	*	*	*	*	*	*	*	*	*	9
Minderhoud C et al. [29] (2022)	*	*	*	*			*	*	*	7

Supplementary Table 4. Quality evidence assessment according the GRADEpro tool (available at https://www.gradepro.org).

Certainty assessment							№ of patients		Effect		Certainty	Importance
№ of studies	Study design	Risk of bias	Inconsistency	Indirectness	Imprecision	Other considerations	CYP2C19 LOF alleles carriers	CYP2C19 LOF alleles non-carriers	Relative (95% CI)	Absolute (95% CI)		
**Stroke**												
6	observational studies	not serious	not serious	not serious	serious	none	42/373 (11.3%)	54/1018 (5.3%)	**RR 1.68** (1.04 to 2.71)	**36 more per 1.000** (from 2 more to 91 more)	⨁⨁⨁◯ Moderate	CRITICAL
**Composite outcome events**												
4	observational studies	not serious	serious	not serious	serious	none	28/226 (12.4%)	62/616 (10.1%)	**RR 1.15** (0.58 to 2.28)	**15 more per 1.000** (from 42 fewer to 129 more)	⨁◯◯◯ Very low	CRITICAL
**Bleeding**												
3	observational studies	not serious	not serious	not serious	serious	none	8/255 (3.1%)	31/798 (3.9%)	**RR 0.84** (0.38 to 1.86)	**6 fewer per 1.000** (from 24 fewer to 33 more)	⨁◯◯◯ Very low	IMPORTANT

CI: confidence interval; **RR:** risk ratio
